# Does restricted distribution limit access and coverage of yellow fever vaccine in the United States?

**DOI:** 10.3201/eid0404.980427

**Published:** 1998

**Authors:** T P Monath, J A Giesberg, E G Fierros

**Affiliations:** OraVax Inc., Cambridge, Massachusetts, USA.


### Does Restricted Distribution Limit Access and Coverage of Yellow Fever Vaccine in the United States?

Yellow fever, the original "viral hemorrhagic fever," is a mosquito-borne infection characterized by hepatic, renal, and myocardial injury, bleeding, and a high case-fatality rate. The disease occurs in tropical South America and sub-Saharan Africa. Maintained by zoonotic transmission between sylvan mosquitoes and nonhuman primates, the virus cannot be eradicated, and prevention of human infection requires high vaccination coverage. Recently the incidence of yellow fever has increased dramatically (1), with 23,543 cases and 6,421 deaths officially reported to the World Health Organization (WHO) between 1985 and 1996. The true incidence is believed to far exceed the reported incidence. Yellow fever remains a public health problem because of failure to implement effective immunization, particularly in Africa.

The recent upsurge in yellow fever activity has been associated with an increased risk for infection among unimmunized travelers. In 1996, two tourists infected in the Amazon region of Brazil died after returning to the United States (2) and Switzerland (3). Fatal and other cases in tourists were also reported in previous years (4-8). Other cases were likely missed, misdiagnosed as viral hepatitis, treated abroad, or not reported.

Since viremic humans are the source of infection for the epidemic mosquito vector *Aedes aegypti*, unimmunized travelers may import yellow fever to *Ae. aegypti*-infested regions, including the United States, West Indies, Central America, Mediterranean region, and Asia. The expansion of international travel, including adventure travel to remote and tropical areas, has increased exposure to yellow fever and other exotic infections (9). Each year eight to nine million tourists arrive in yellow fever-endemic countries in South America and Africa (10,11); the number of tourists visiting regions within countries in which yellow fever transmission occurs is unknown but probably exceeds three million. In the United States, 150,000 to 300,000 civilian travelers are immunized annually (12)—likely only a fraction of the population requiring the vaccine. However, few data exist on vaccine coverage in travelers to yellow fever–endemic regions. One survey in the United States found that 30% of travelers to developing countries between 1980 and 1986 received yellow fever vaccine (13). Yellow fever vaccine coverage of European travelers varied from 0% to 90% (14,15).

WHO and the Centers for Disease Control and Prevention strongly recommend vaccination for travel outside urban areas in the yellow fever–endemic zone (16,17). Yellow fever 17D, a live, attenuated, vaccine developed in 1936, has been used in more than 300 million persons and has a remarkable record of safety, tolerance, and efficacy (18). Immunity develops rapidly after a single dose and is extraordinarily durable, probably lifelong. Yellow fever vaccine can be administered simultaneously with most other vaccines or immune serum globulin recommended for international travel. Cost of the vaccine is approximately $48 for a single-dose vial in the United States. Thus, the attributes of the vaccine itself do not constitute a barrier to the immunization of travelers.

Yellow fever is the only human vaccine subjected to regulations regarding distribution. The International Health Regulations (19) require that yellow fever vaccine be given in approved vaccinating centers, listed in a 1991 WHO publication (20) and updated in the WHO Weekly Epidemiological Record. The restriction was instituted to ensure that the relatively thermolabile vaccine is properly stored and delivered within the specified time after reconstitution and that a signed vaccination certificate is properly completed (specifying the date of immunization and the vaccine lot) and bears an official stamp. Only authorized medical facilities may order vaccine directly from the manufacturer, and the vaccine is not available through the normal distribution channels for drugs and biologic products.

In the United States, registration and approval of yellow fever vaccinating centers has been delegated by the U.S. Public Health Service to the state health departments. With the expansion of international travel in recent years and a growing number of travel clinics, this system has become unwieldy, and its listing of active centers is inaccurate.

### The Problem, the Risks, a Solution

The United States currently has 800 to 1,000 yellow fever vaccinating centers. Many are county health departments or clinics specializing in travel medicine. However, most travelers first seek pretravel medical advice from their primary-care providers. Immunizations and prophylactic measures against many travel-related threats, such as hepatitis A, typhoid, diphtheria, and measles, are commonly given by primary-care providers; even more specialized travel vaccines, such as rabies, meningococcal, and Japanese encephalitis vaccines, are readily available to general practitioners. Specific recommendations for the use of travel vaccines are in publications (19,20) and on the Internet.

Yellow fever is the only travel vaccine for which the primary-care physician must refer the patient to another provider. Compliance with recommendations for immmunization is affected by multiple factors (21), including inconvenient access to a vaccine. A Tennessee resident who died of yellow fever in 1996 was not vaccinated before going to Brazil because the official vaccinating center nearest his home was 25 miles away (2). In contrast, he had received other medical advice, hepatitis A vaccine, and a prescription for malaria chemoprophylaxis from his primary-care physician. Since requests for yellow fever vaccine may be few, most physicians do not initiate the complicated process of obtaining authorization as a designated vaccinating center. In the United Kingdom, however, where general practitioners have become proactive in providing travel advice to their patients, the number of physicians who obtained yellow fever vaccinating center status rose from 181 to 1,231 between 1990 and 1992 (22).

The disadvantages of general practitioners' engaging in travel medicine, including the immunization of travelers against exotic infections and prescription of malaria chemoprophylaxis, have been examined in several studies, which suggest that improvements are needed in the quality of advice provided to patients (23,24). Among Swiss and German general practitioners, only 11% and 1%, respectively, provided correct recommendations to travelers to Thailand and Kenya regarding malaria prophylaxis and vaccinations (25). County health departments in the United States responsible for yellow fever vaccination have limited expertise in travel medicine. While extending the distribution of yellow fever vaccine to primary health-care providers would increase access to the vaccine and improve coverage, additional steps are required to improve the quality of medical advice by giving practitioners accurate and simple information to guide use of the vaccine. Public health department nurses and inspectors in Ontario, Canada, provided correct recommendations for yellow fever vaccination 96% of the time (26), reflecting the value of education in this field.

Travelers requiring yellow fever and other live virus vaccines face special problems. The current recommendation is for either simultaneous administration or separation of the vaccines by 4 weeks to avoid potential interference (16). Compliance with this recommendation is difficult, and the recommendation is probably ignored when the patient receives some vaccines from the primary-care physician and yellow fever vaccine at a designated center. The recommendation provides a further incentive for a single health professional to administer a schedule of immunizations appropriate for the individual traveler.

We sampled 15 states, which contained a very high proportion of the U.S. population (44%) and were representative with respect to geography, urban-rural distribution, and race. Although we could not estimate whether these states were different from the United States as a whole with respect to international travel, we believe that it is reasonable to extrapolate the findings with respect to access to vaccinating centers to the general population. We found that the current system of designated yellow fever vaccinating centers in the United States provides reasonable access to approximately 95% of the population, where "reasonable access" is defined as residence within a 25-mile radius of the vaccinating center (Table). Although this would appear sufficient, in fact there may be substantial obstacles—from the perspective of the patient—to utilization of centers within geographic proximity (the 25-mile catchment area), including extra costs, inconvenience of access to centers within congested urban areas, and unfamiliarity with the provider. Approximately 5% to 6% of the total U.S. population (12 to 14 million) resides in rural areas that are relatively inaccessible to vaccinating centers (Table). In some states, such as Montana, Wyoming, Oklahoma, and Vermont, approximately 25% of the population resides outside 25-mile catchment areas, and very long distances must be traveled to reach a center (Figure), with the result that only highly motivated patients are vaccinated. Although it is possible that urban

Table. Populations (1990 census[a]) inside and outside 25-mile radius ‘catchment areas’ of intrastate authorized yellow fever vaccinating centers

| | | Population x10$^3$ | | |
|---|---|---|---|---|
| Region | State | Total | Inside catchment area (%) | Outside catchment area (%) |
| East | New York | 17,991 | 17,679 (98.3) | 312 (1 .7) |
| | Massachusetts | 6,016 | 5,965 (99.2) | 51 (0.8) |
| | Connecticut | 3,287 | 3,287 (100) | 0 (0) |
| | Vermont | 563 | 418 (74.2) | 145 (25.8) |
| South | Florida | 12,938 | 12,410 (95.9) | 528 (4.1) |
| | Louisiana | 4,220 | 3,767 (89.3) | 453 (10.7) |
| Central | Illinois | 11,431 | 10,125 (88.6) | 1,306 (11.4) |
| | Wisconsin | 4,892 | 4,331 (88.5) | 561 (11.5) |
| | Nebraska | 1,578 | 1,293 (81.9) | 285 (18.1) |
| | Oklahoma | 3,146 | 2,317 (73.6) | 829 (26.4) |
| Mountain | Montana | 799 | 595 (74.5) | 204 (25.5) |
| | Wyoming | 454 | 350 (77.1) | 104 (22.9) |
| Pacific | California | 29,758 | 29,138 (97.9) | 620 (2.1) |
| | Oregon | 2,842 | 2,676 (94.2) | 166 (5.8) |
| | Hawaii | 1,108 | 983 (88.7) | 125 (11.3) |
| 15 states | | 101,023 | 95,334 (94.4) | 5,689 (5.6) |

[a]U.S. Bureau of the Census, 1990 Decennial Census.

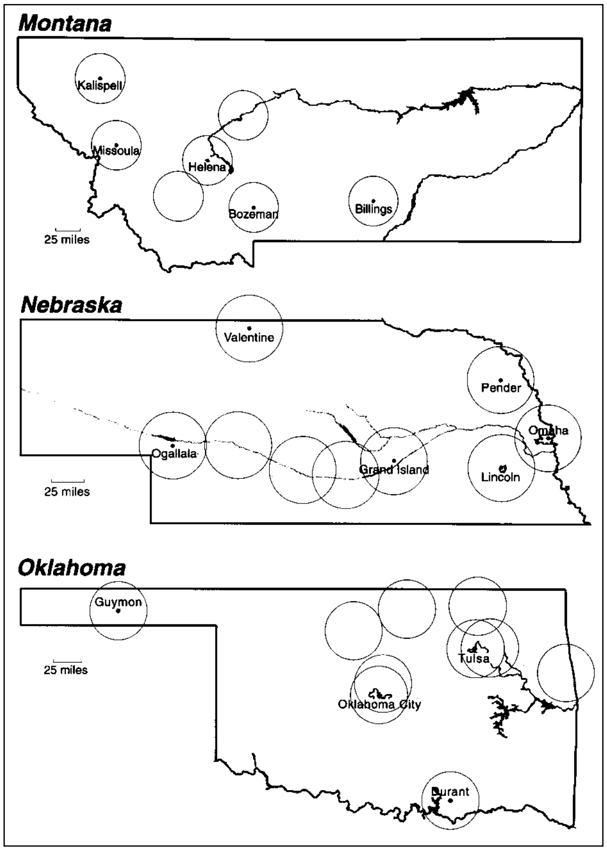

Figure. Location of yellow fever vaccinating centers in three western states. The circles indicate the area within a 25-mile radius from each center (catchment areas). Only names of selected locations are shown.

residents with better access to vaccinating centers are more likely to internationally travel than rural residents, our analysis clearly demonstrated that restricted access to yellow fever vaccine by populations in rural or sparsely populated areas.

In the past, a principal concern underlying the restricted distribution of yellow fever vaccine was the thermolability of the vaccine and the need to ensure its proper storage and handling. However, other vaccines in wide use have similar or even more stringent storage requirements. Tetanus toxoid must be stored refrigerated away from the freezer compartment, measles-mumps-rubella must be stored refrigerated and protected from light, and varicella vaccine must be stored frozen. Yellow fever vaccine manufacturers now include stabilizers in the product that render the lyophilized vaccines quite resistant to degradation (27-29). Yellow fever vaccine is stored in the refrigerator at the point of use, as are most other vaccines. Yellow fever vaccine is discarded 1 hour after reconstitution; in contrast, varicella vaccine must be discarded within 30 minutes. Thus, the storage and handling of yellow fever vaccine do not pose special difficulties.

The International Health Regulations, adopted nearly 30 years ago (19), require a valid certificate of vaccination only for yellow fever (17,19). This regulation recognizes the risk for importation and spread of yellow fever across international boundaries by viremic travelers. However, enforcement is problematic and lax. For example, in the Calcutta airport, closure of the health inspection desk in 1986 virtually eliminated verification of vaccination status of disembarking passengers (30). A number of yellow fever–receptive (*Ae. aegypti*-infested) countries, including the United States, do not require vaccination certificates (16,17), and many countries require but do not enforce the inspection of certificates. The United States abandoned inspection of vaccination certificates (because of the high cost and questionable public health value of yellow fever quarantine regulations) in favor of a system of surveillance and optimization of health information to the traveling public. The difficulty in controlling yellow fever vaccination through designated centers and the lack of a clear justification on the basis of thermostability of the vaccine led a WHO working group on revisions to the International Health Regulations to recommend reconsideration of designation procedures of vaccination centers (31). We think the link between the requirement for certification of yellow fever vaccination imposed by some national health authorities and the distribution of vaccine through designated centers should be the focus of future discussions between parties to the International Health Regulations. Certification of vaccination could be provided by any licensed physician, as was the case for smallpox and cholera vaccines at the time that these were required for international travel. The blank certificate (available from the Government Printing Office) could, for example, be shipped together with the vaccine from the manufacturer or distributor to the physician. A change would be required in the International Health Regulations to allow validation of the certificate by physician signature alone without application of the uniform stamp currently used on the certificate.

The risk of acquiring yellow fever during travel is related to the level of virus transmission at the time and in the location visited. During yellow fever epidemics, the risk to an unimmunized person is very high, even during a short visit to an endemic-disease area. In recent outbreaks affecting indigenous populations in West Africa, the incidence of infection during the 3-month epidemic period was 20% to 30% (32,33). The risk of developing severe disease with jaundice if infected has been estimated at 1:3.8 to 1:7.4 and the risk of dying if ill with jaundice is 20% to 60% (18). In areas where the indigenous population is protected by vaccination, yellow fever virus may circulate silently between monkeys and mosquitoes without any reported human cases as a guide to risk. Moreover, the southern United States is infested with *Ae. aegypti*, and importation of yellow fever poses a considerable threat (34,35). The potential for importation by persons infected with yellow fever has increased with the establishment of direct links between the United States and disease-endemic areas, such as Manaus, Brazil, and Iquitos, Peru (9). These factors provide a strong rationale for immunizing all persons who visit disease-endemic zones.

The current system of designated yellow fever vaccinating centers and relative inaccessibility to yellow fever vaccine are impediments to full vaccination coverage of travelers. Extension of vaccinating privileges to primary-care providers, together with clear and concise information regarding recommendations for use of the vaccine, may improve protection of travelers at risk and enhance the barrier to importation of the disease.

**Thomas P. Monath,* Judith A. Giesberg,† and Edward Garcia Fierros,‡**
*OraVax Inc., Cambridge, Massachusetts, USA and Harvard School of Public Health, Boston, Massachusetts, USA; †Boston College, Chestnut Hill, Massachusetts, USA; ‡Harvard Graduate School of Education, Cambridge, Massachusetts, USA

### References

1. Robertson SE, Hull BP, Tomori O, Bele O, LeDuc JW. Yellow fever. A decade of reemergence. JAMA 1996;276:1157-62.
2. McFarland JM, Baddour LM, Nelson, Elkins SK, Craven RB, Cropp BC, et al. Imported yellow fever in a United States citizen. Clin Infect Dis 1997;25:1143-7.
3. Barros ML, Boecken G. Jungle yellow fever in the central Amazon. Lancet 1996;348:969.
4. Bendersky N, Carlet J, Ricomme JL, Souied G, Belaiche J, Lange F, et al. Deux cas de fièvre jaune observés en France et contractés au Sénégal. Aspects épidémiologiques et cliniques. Bull Soc Pathol Exot 1980;73:54-61.
5. Digoutte JP, Plassart H, Salaun JJ, Heme G, Ferrara L, Germain M. A propos de trois cas de fièvre jaune contractée au Sènègal. Bull World Health Organ 1981;59:759-66.
6. World Health Organization. Yellow fever in 1981. Wkly Epidemiol Rec 1982;59:297-301.
7. World Health Organization. Yellow fever in 1985. Wkly Epidemiol Rec 1986;61:377-80.
8. Nolla-Salas J, Sadalls-Radresa J, Bada JL. Imported yellow fever in vaccinated tourist. Lancet 1989:2:1275.
9. Monath TP. Epidemiology of yellow fever: current status and speculations on future trends. In: Saluzzo J-F, Dodet B, editors. Factors in the emergence of arbovirus diseases. Paris: Elsevier 1997. p. 143-56.
10. Steffen R. Hepatitis A in travelers: the European experience. J Infect Dis 1995;171 Suppl:S24-8.
11. World Health Organization. World Health Report 1996. Geneva: The Organization; 1996. p. 137.
12. Centers for Disease Control and Prevention. Biologics surveillance. Atlanta (GA): The Centers; 1996. Report No. 94.

13. Jong EC, Gurung K. Observations on obtaining immunizations for international travel and the need for routine immunizations. In: Steffen R, Lobel HO, Haworth J, Bradley DJ, editors. Travel medicine. Proceedings of the 1st International Conference on Travel Medicine; 1988 Apr 5-8; Zurich, Switzerland. Berlin: Springer Verlag; 1989. p. 217-9.
14. Steffen R, Raeber PA. Vaccinations pour les voyageurs internationaux. World Health Stat Q 1989;42:85-9.
15. Prazuck T, Semaille C, Defayolle M, Bargain P, Clerel M, Lafaix C, et al. Statut vaccinal des voyageurs francais et européens: etude de 9156 sujets au départ de Paris vers 12 destinations tropicales. Rev Epidemiol Sante Publique 1998;46:64-72.
16. Centers for Disease Control and Prevention. Health information for international travel 1996-97. Atlanta (GA): U.S. Department of Health & Human Services; 1997. p. 1-208.
17. World Health Organization. International travel and health. Vaccination requirements and health advice. Geneva: The Organization; 1997. p. 104.
18. Monath TP. Yellow fever. In: Plotkin SA, Mortimer EA, Orenstein W, editors. Vaccines, 3rd Ed. Philadelphia: WB Saunders. In press 1998.
19. World Health Organization. International Health Regulations (1969) 3rd annotated ed. Geneva: The Organization; 1983.
20. World Health Organization. Yellow-fever vaccinating centres for international travel. Geneva: The Organization; 1991.
21. Keystone JS, Reid D. Compliance with travel health recommendations. Chapter 24. In: DuPont HL, Steffen R, editors. Textbook of travel medicine and health. Zurich, Switzerland: BC Decker; 1997. p. 282-6.
22. Sloan DSG. Travel medicine and general practice: a suitable case for audit? Lancet 1993;307:615-8.
23. Usherwood V, Usherwood TP. Survey of general practitioners' advice for travellers to Turkey. Journal of the Royal College of General Practitioners 1989;39:148-50.
24. Mott A, Kinnersley P. Overprescription of cholera vaccine to travellers by general practioners. BMJ 1990;300:25-6.
25. Hatz C, Krause E, Junghans S. Travel advice—a study among Swiss and German general practitioners. In: Abstracts of the 4th International Conference on Travel Medicine; 1995 Apr 23-29; Acapulco, Mexico. Abstract 24.
26. MacPherson DW, Stephenson BJ, Keystone JS, Sawyer L. Travel health information by public health departments: a telephone survey. In: Abstracts of the 4th International Conference on Travel Medicine; 1995 Apr 23-29; Acapulco, Mexico. Abstract 23.
27. Monath TP. Stability of yellow fever vaccine. Dev Biol Stand 1996;87:219-25.
28. Burfoot C, Young PA, Finter NB. The thermal stability of stabilized 17D yellow fever vaccine. Journal of Biological Standards 1977;5:173-9.
29. Barme M, Bronnert C. Thermostabilisation du vaccin antiamaril 17D lyophilisé. I. Essai de substances protectrices. Journal of Biological Standards 1984;12:435-42.
30. Sinha MK, Majumder NM. Will the present health check-up system invite yellow fever in India? Indian J Public Health 1990;34:119-21.
31. World Health Organization. The international response to epidemics and applications of the International Health regulations. Report of a WHO informal consultation. Geneva: The Organization; 1996 WHO Document WHO/EMC/IHR/96.1.
32. Monath TP, Craven RB, Adjukiewicz A, Germain M, Francy DB, Ferrara L, et al. Yellow fever in the Gambia, 1978-1979: epidemiologic aspects with observations on the occurrence of Orungo virus infections. Am J Trop Med Hyg 1980;29:912-28.
33. DeCock KM, Monath TP, Nasidi A, Tukei PM, Enriquez J, Lichfield P, et al. Epidemic yellow fever in Eastern Nigeria, 1986. Lancet 1988;19:630-3.
34. Sawyer WA. Public health implications of tropical and imported diseases. Yellow fever and typhus and the possibility of their introduction into the United States. Am J Public Health 1944;34:7-14.
35. Hughes JH, Porter JE. Measures against yellow fever entry into the United States. Public Health Rep 1958;73:1101-6.
